# Morphostatic Speciation within the Dagger Nematode *Xiphinema hispanum-*Complex Species (Nematoda: Longidoridae)

**DOI:** 10.3390/plants9121649

**Published:** 2020-11-26

**Authors:** Antonio Archidona-Yuste, Ruihang Cai, Carolina Cantalapiedra-Navarrete, José A. Carreira, Ana Rey, Benjamín Viñegla, Gracia Liébanas, Juan E. Palomares-Rius, Pablo Castillo

**Affiliations:** 1 Department of Ecological Modelling, Helmholtz Centre for Environmental Research—UFZ, Permoserstrasse 15, 04318 Leipzig, Germany; 2 Instituto de Agricultura Sostenible (IAS), Consejo Superior de Investigaciones Científicas (CSIC), Avda. Menéndez Pidal s/n, 14004 Córdoba, Spain; ruihangcai@163.com (R.C.); carocantalapiedra@hotmail.com (C.C.-N.); palomaresje@ias.csic.es (J.E.P.-R.); p.castillo@csic.es (P.C.); 3 Laboratory of Plant Nematology, Institute of Biotechnology, College of Agriculture and Biotechnology, Zhejiang University, Hangzhou 310058, China; 4 Departamento de Biología Animal, Biología Vegetal y Ecología, Universidad de Jaén, Campus Las Lagunillas, 37724 Jaén, Spain; jafuente@ujaen.es (J.A.C.); bvinegla@ujaen.es (B.V.); gtorres@ujaen.es (G.L.); 5 Departamento de Biogeografía y Cambio Global, Museo Nacional de Ciencias Naturales-CSIC, José Abascal 2, 28006 Madrid, Spain; arey@mncn.csic.es

**Keywords:** Bayesian inference, cryptic species, *coxI*, *D2-D3* expansion domains of *28S* rRNA-gene, integrative taxonomy, principal component analysis

## Abstract

Dagger nematodes of the genus *Xiphinema* include a remarkable group of invertebrates of the phylum Nematoda comprising ectoparasitic animals of many wild and cultivated plants. Damage is caused by direct feeding on root cells and by vectoring nepoviruses that cause diseases on several crops. Precise identification of *Xiphinema* species is critical for launching appropriate control measures. We deciphered the cryptic diversity of the *Xiphinema hispanum*-species complex applying integrative taxonomical approaches that allowed us to verify a paradigmatic example of the morphostatic speciation and the description of a new species, *Xiphinema malaka* sp. nov. Detailed morphological, morphometrical, multivariate and genetic studies were carried out, and mitochondrial and nuclear haploweb analyses were used for species delimitation of this group. The new species belongs to morphospecies Group 5 from the *Xiphinema* non*americanum*-group species. *D2-D3*, *ITS1*, partial *18S*, and partial *coxI* regions were used for inferring the phylogenetic relationships of *X. malaka* sp. nov. with other species within the genus *Xiphinema*. Molecular analyses showed a clear species differentiation not paralleled in morphology and morphometry, reflecting a clear morphostatic speciation. These results support the hypothesis that the biodiversity of dagger nematodes in southern Europe is greater than previously assumed.

## 1. Introduction

Plant-parasitic nematodes (PPN) are characterized by the presence of a stylet used for root tissue penetration, comprise about 15% of the total number of nematode species currently known, of which over 4100 species have been identified as PPN [1,2]. Annual crop losses caused by PPN are estimated to be about 8–15% of total crop production worldwide [3,4]. Accurate identification of PPN is essential for the selection of appropriate control measures against plant pathogenic species, as well as for a reliable method allowing distinction between species under quarantine or regulatory strategies and a better understanding of their implications in pest control and soil ecology [5,6]. PPN species have been defined historically based on morphological characteristics [7,8]. However, the adoption of molecular techniques in nematode taxonomy has revealed unexpected genetic diversity within species throughout the phylum Nematoda [9]. This has been especially accurate for the family Longidoridae, a large group of ectoparasitic nematodes feeding from the root tip zone to the hairy root region, and characterized by a substantial intra and interspecific homogeneity of the morphometric characters used for species discrimination [1,6,10,11]. Use of molecular data in species identification of dagger and needle nematodes over the last three decades has indicated that many widespread species actually comprise multiple genetically divergent and morphologically similar cryptic species [6,11,12,13]. Complexes of cryptic species often result from nonecological speciation in which diversification is not accompanied by apparent ecological or morphological separation in traditional quantitative traits [14].

The genus *Xiphinema* is one of the most diversified group species of longidorid nematodes with more than 280 valid species [5,6,11,15]. The ecological and phytopathological importance of this group of nematodes lies in its wide range of host plants and cosmopolitan distribution [5,11], but some species of this genus are vectors of several important plant viruses (genus *Nepovirus*, family Comoviridae) that cause significant damage to a wide range of crops [10,16]. Considering the great diversity of this group, the genus *Xiphinema* was divided into two different species groups [5,17,18]: (i) the *Xiphinema americanum*-group comprising a complex of about 60 species [15,17]; and (ii) the *Xiphinema* non*americanum*-group which comprises a complex of more than 220 species [5,6,19]. Later, this group was divided into eight morphospecies groups for helping identification [18]. However, some cryptic species and species complexes within *Xiphinema* have been recently revealed based on integrative taxonomical approaches, including morphometric multivariate methods, genetic analyses based on ribosomal and mitochondrial DNA (rDNA and mtDNA, respectively) and species delimitation (haplonet tools) [6,11,20,21]. A paradigmatic example of these species complexes comprises the *Xiphinema hispanum*-complex, *viz*. didelphic *Xiphinema* species from the Iberian Peninsula characterized by a rounded tail in females with or without an inconspicuous bulge projecting slightly ventrally and a uterus showing spiniform structures [22]. The cryptic diversity of this species complex has been deciphered by our team over the last ten years applying integrative taxonomical approaches that allowed us to verify these species as valid, and the recent description of a new species, *X. subbaetense* [11,20]. Recent studies on this species complex clearly separated three species (*X. adenohystherum*, *X. hispanum* and *X. subbaetense*) revealing high levels of genetic diversity within them that showed little morphological differentiation [11]. In new nematode surveys carried out in natural areas in the provinces of Málaga and Almería, Andalusia, southern Spain, we have detected nine unidentified *Xiphinema* isolates resembling *X. hispanum*-complex morphology. Detailed morphological and morphometrical observations using light microscopy indicated that these isolates appeared undistinguishable from *X. hispanum* complex species, a fact which prompted us to undertake comprehensive multivariate and genetic analyses, compared with previous reported data, to decipher this taxonomic conundrum.

Morphostatic evolution can be defined as genetic modifications, and even complete speciation events, which are not reflected in morphology, often being a result of nonadaptive radiation marked by the rapid proliferation of species without ecological differentiation [23,24]. Although no data have yet been specifically mentioned in Nematoda, morphostatic evolution seems not to be a rare phenomenon in longidorids based on the numerous complexes and cryptic species documented [6,11,12,13,15,20,25]. In Longidoridae, it is very common that molecular divergences among species are not reflected in morphological or morphometric traits, which conforms a morphostatic model of evolution with numerous cryptic species within this group [6,11,13,15,20,21,25,26].

In this context, we investigated (1) the existence of a new cryptic species within the *X. hispanum*-complex confirming a morphostatic speciation in this group using an integrative species delineation approach based on multivariate morphometric analysis and haplonet mitochondrial and nuclear haploweb tools; (2) a new species of the genus *Xiphinema* (*Xiphinema malaka* sp. nov.) described through integrative methods based on the combination of morphological, morphometric and molecular data; and (3) phylogenetic analyses based on *D2-D3* expansion domains of the *28S* rRNA gene, *ITS1*, the partial *18S* rRNA gene, and the partial mitochondrial *coxI* gene sequences to clarify the relationships of the new *Xiphinema* species.

## 2. Results

**Figure 1:**
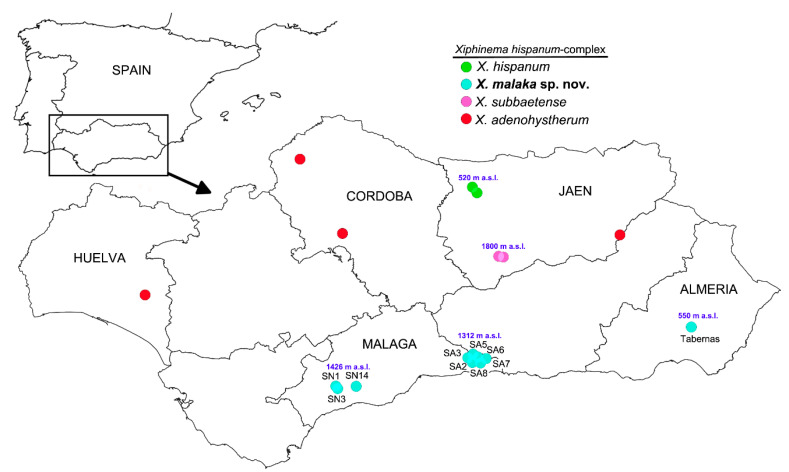
Geographic distribution of *Xiphinema hispanum*-complex species and locations of sampling sites of which the recovered isolates of the new species were characterized morphometrically and molecularly. Arrow indicates the location of Andalusia in the Iberian Peninsula.

Species boundaries within the *Xiphinema* complex included in this research (Figure 1) were based on the integrative application of morphological, morphometric and molecular methods to unravel potential cryptic species diversity (Table 1). Species delimitation was carried out using two independent approaches based on morphometric (multivariate analysis) and molecular data using ribosomal and mitochondrial sequences (haplonet). Multivariate morphometric and haplonet methods were performed on the nine studied isolates including previous isolates from the *X. hispanum*-complex to verify species identifications. The integration of this procedure with the analysis of nematode morphology allowed us to verify *Xiphinema malaka* sp. nov. as a valid new species within the *X. hispanum* cryptic complex. Additionally, we maintained a consensus approach for the different species delimitation methods, including concordant results in phylogenetic trees inferred from nuclear and mitochondrial markers and/or different morphological or morphometric characteristics.

### 2.1. Multivariate Morphometric Analysis

**Figure 2:**
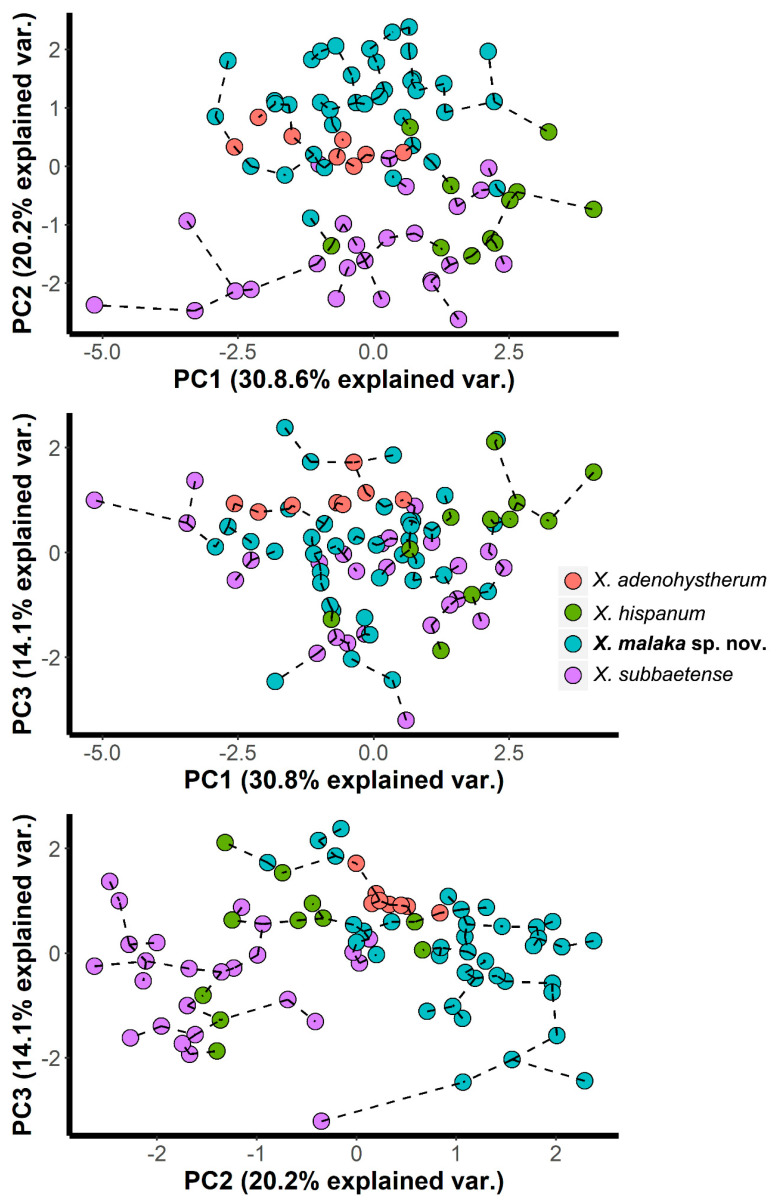
Principal component analysis on morphometric characters to characterize *Xiphinema hispanum-*complex with a superimposed minimum spanning tree (based on Euclidean distance).

In principal component analysis (PCA), the first three components (sum of squares (SS) loadings > 1) accounted for 65.1% of the total variance in the morphometric characteristics of the *X. hispanum-*complex (Table 2). The eigenvalues for each character were used to interpret the biological meaning of the factors. First, the principal component 1 (PC1) was mainly dominated by a stylet with a high positive correlation (eigenvalue = 0.523). PC2 was mainly dominated by high negative correlation for the vulva position (eigenvalue = −0.547) as well as a high positive correlation for the a ratio (eigenvalue = 0.482) (Table 2). This component was, therefore, related with the overall nematode size and shape. Finally, PC3 was mainly dominated by the highest positive correlation found for the c’ ratio and lower, but also high, positive correlation for the hyaline region length (eigenvalues = 0.774 and 0.458, respectively). This component was then related with tail shape. Overall, these results suggest that all of the extracted PCs were related to the overall size and shape of nematode isolates. The results of the PCA were represented graphically in Cartesian plots in which isolates of the *X. hispanum-*complex were projected on the plane of the *x*- and *y*-axes, respectively, as pairwise combinations of components 1 to 3 (Figure 2). In the graphic representation of the *X. hispanum-*complex, and with the exception of *X. adenohystherum*, we observed that the specimens of all species were projected showing an expanded distribution along the plane for all the projected combinations of the components. One reason might be the wide morphometric variation detected in these species (Table 3 and Table 4) [6,11]. As a consequence, we did not detect a clear separation amongst species within the *X. hispanum-*complex, all the specimens being projected at random for all the projected combinations. These patterns suggest a clear example of morphostatic speciation within the *X. hispanum-*complex. However, it should be noted that when projected on the plane of the combinations of PC1-2 and PC2-3, almost all specimens of *X. malaka* sp. nov. and *X. subbaetense* were separated among them (Figure 2). This graphical separation was shown by the projection of PC2 (dominated by the V and a ratios). This graphical separation is due to the variation found in the ratio a among these species, as pointed out below. A minimum spanning tree (MST) superimposed on the plot of the first three principal components showed the same patterns observed with PCA, that is, not clear separation amongst species within the *X. hispanum*-complex (Figure 2). 

### 2.2. Mitochondrial Haplonet and Nuclear Haploweb Networks

**Figure 3:**
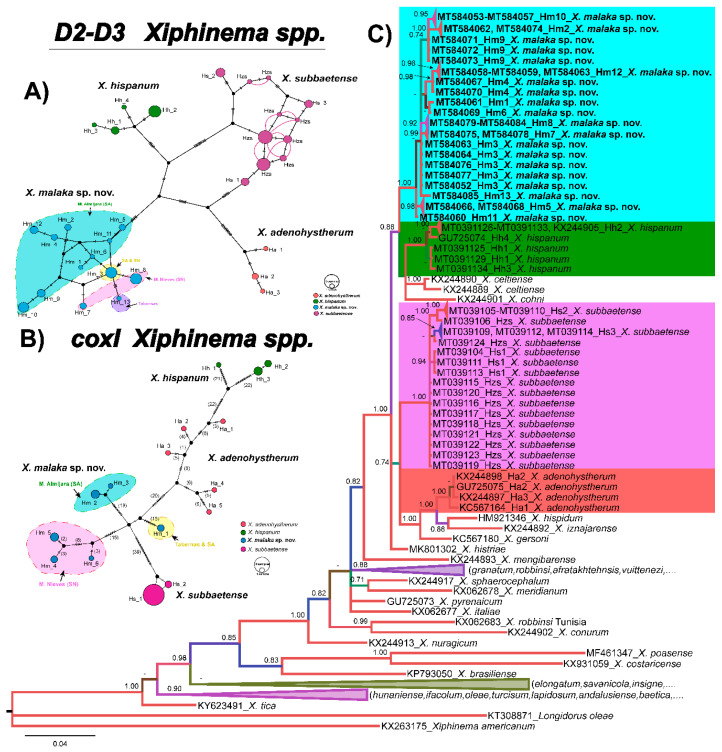
(**A**). Construction of *D2-D3* haploweb of *Xiphinema malaka* sp. nov. (**B**). *coxI* haplonet of *Xiphinema malaka* sp. nov. Coloured circles represent haplotypes and their diameter are proportional to the number of individuals sharing the same haplotype. Black short lines on the branches indicate the number of mutated positions in the alignment that separate each haplotype. Co-occurring haplotypes are enclosed in black dashes. (**C**). Phylogenetic relationships within the genus *Xiphinema*. Bayesian 50% majority rule consensus tree as inferred from D2 and D3 expansion domains of *28S* rRNA sequence alignment under the general time-reversible model of sequence evolution with correction for invariable sites and a gamma-shaped distribution (GTR + I + G) +. Posterior probabilities more than 0.70 are given for appropriate clades. Newly obtained sequences in this study are shown in bold. Scale bar = expected changes per site. Some branches were collapsed for improving readability of *Xiphinema* species. Abbreviations: Ha = *X. adenohystherum* haplotypes; Hh = *X. hispanum* haplotypes; Hm = *X. malaka* sp. nov. haplotypes; Hs = *X. subbaetense* haplotypes; Hzs = *X. subbaetense* heterozygous specimens. SA = Mountain of Almijara and Tejeda; SN = Mountain of Nieves.

Species delimitation using haplonet methods in *X. hispanum-*complex species contained 75 sequences (35 sequences from *X. malaka* sp. nov., four sequences from *X. adenohystherum*, 13 sequences from *X. hispanum*, and 23 sequences from *X. subbaetense*) with 13, 3, 4, and 3 different haplotypes and several heterozygous individuals, respectively (Table 1, Figure 3A). The TCS haplotype analysis inferred from the *D2-D3* region showed four well-differentiated haplogroups corresponding to four different main lineages (*X. adenohystherum*, *X. hispanum*, *X. malaka* sp. nov., and *X. subbaetense*) (Figure 3A). *Xiphinema malaka* sp. nov. comprised a higher diversity in Mountain Almijara (SA, with nine haplotypes) than that detected in Mountain Nieves (SN 2 haplotypes), one haplotype in Tabernas, and one haplotype (Hm3) jointly detected in SA and SN (Figure 3A). 

However, in *coxI* haplonet (Figure 3B), six different haplotypes of *X. malaka* sp. nov. were detected, three in SN and three in SA. One from SA shared the same haplotype with the Tabernas isolate, and this haplotype kept a far molecular distance with the other two haplotypes from SA. It was worth noting that the number of *D2-D3* haplotypes of *X. malaka* sp. nov. was higher than *coxI* haplotypes (13 vs. 6), but there were more mutations between these *coxI* haplotypes than *D2-D3* haplotypes (Figure 3A,B). Besides, *X. subbaetense* also comprised more haplotypes in the *D2-D3* haplonet than the *coxI* haplonet (11 vs. 2); the situation of *X. hispanum*, *X. adenohystherum* were the same as previously described by Cai et al. [11].

### 2.3. Molecular Characterization

The amplification of *D2-D3* expansion domains of *28S* rRNA, *ITS1* rRNA, the partial *18S* rRNA, and partial *coxI* genes, yielded single fragments of ~900 bp, 1100 bp, 1800 bp, and 500 bp, respectively, based on gel electrophoresis. *D2-D3* for *X. malaka* sp. nov. (MT584052–MT584085) showed a low intraspecific variability with 1–7 different nucleotides and 0 indels (99% similarity). The molecular diversity of this marker within SA (1–7 nucleotides, 0 indels) and SN (2–3 nucleotides, 0 indels) isolates was similar among them and differed from the closest related species, *X. hispanum* (KX244905, MT039125–MT039134) by 20–21 different nucleotides and 1–2 indels (97% similarity), *X. subbaetense* (MT039104–MT039124) by 22–25 different nucleotides and 2–3 indels (97% similarity), and from *X. adenohystherum* (KC567164, KX244898, GU725075, KX244897) by 29–42 different nucleotides and 3 indels (96% similarity).

The *ITS1* region for *X. malaka* sp. nov. showed an intraspecific variability with 26–39 different nucleotides and 4–10 indels (96%–98% similarity). The molecular diversity of this marker within SA (18–24 nucleotides, 1–4 indels) and SN (26–29 nucleotides, 4 indels) isolates was also similar among them. *ITS1* for *X. malaka* sp. nov. (MT584088-MT584099) differed from the closest related species, *X. subbaetense* (MT026293–MT026295) by 132–136 different nucleotides and 28–29 indels (88% similarity), *X. hispanum* (GU725061) by 84–142 different nucleotides and 22–38 indels (87–90% similarity), and from *X. adenohystherum* (GU725063, MT584100–MT584102) by 133–139 different nucleotides and 40–45 indels (87–88% similarity).

For the *18S* rRNA, two new identical sequences for *X. malaka* sp. nov. (MT584086–MT584087) were obtained in this study and both of them showed very high similarity values with other accessions from *Xiphinema* spp. deposited in GenBank, being 98–99% similar. From the closet related species they differed by 1–2 nucleotides and 0 indels from *X. subbaetense* (MT039135–MT039140), *X. adenohystherum* (GU725084) by two nucleotides different and 0 indels, and *X. hispanum* (GU725083) by one nucleotide different and 0 indels. Finally, thirteen new *coxI* sequences for *X. malaka* sp. nov. (MT580263–MT580274) were deposited in GenBank in this study. This gene showed an intraspecific variability with 3–48 different nucleotides and 0 indels (88–99% similarity). The molecular diversity of this marker within SA (0–2 nucleotides, 0 indels) and SN (0–9 nucleotides, 0 indels) isolates was similar among them. *coxI* for *X. malaka* sp. nov. (MT580263–MT580274) differed from the closest related species, *X. subbaetense* (MT040280–MT010300) by 59–66 different nucleotides and 0 indels (82% similarity), *X. hispanum* (KY816614, MT040301-MT040305) by 51–78 different nucleotides and no indels (78–81% similarity), and from *X. adenohystherum* (KY816588–KY816592) by 58–65 different nucleotides and no indels (82–85% similarity).

### 2.4. Phylogenetic Relationships

**Figure 4:**
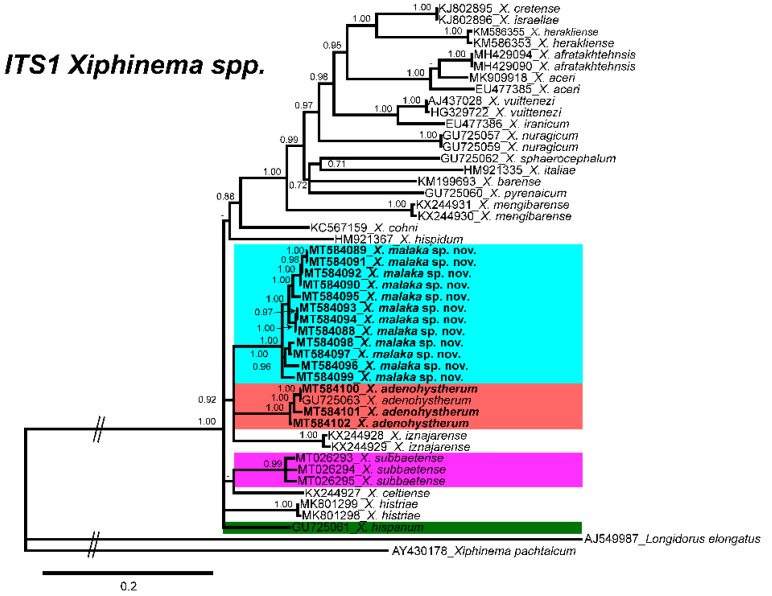
Phylogenetic relationships of *Xiphinema malaka* sp. nov. within the genus *Xiphinema*. Bayesian 50% majority-rule consensus trees as inferred from *ITS1* sequence alignments under transition model with a proportion of invariable sites and a rate of variation across sites (TIM2 + I + G). Posterior probabilities more than 70% are given for appropriate clades. Newly obtained sequences in this study are in bold letters, and each colour is associated with each species of the complex.

**Figure 5:**
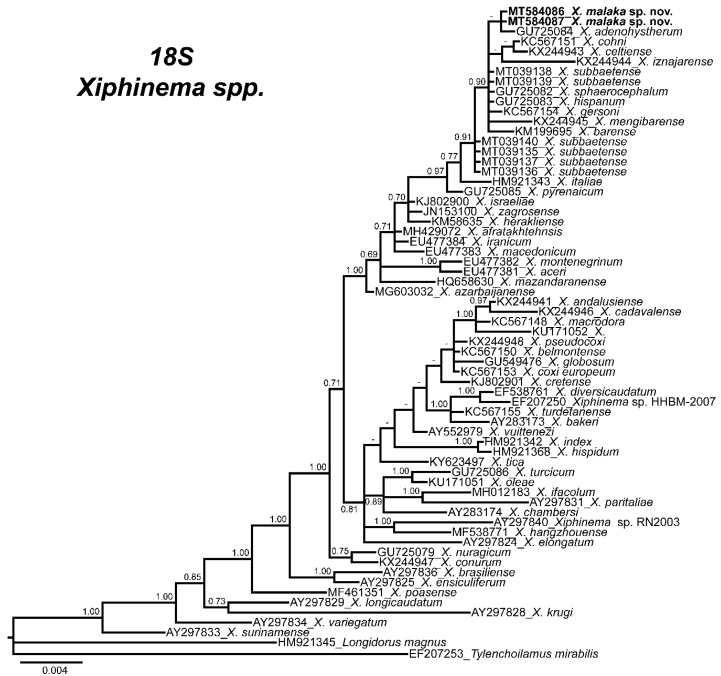
Phylogenetic relationships of *Xiphinema malaka* sp. nov. within the genus *Xiphinema*. Bayesian 50% majority-rule consensus trees as inferred from *18S* sequence alignments under the GTR + I + G model. Posterior probabilities more than 70% are given for appropriate clades. Newly obtained sequences in this study are in bold letters.

**Figure 6:**
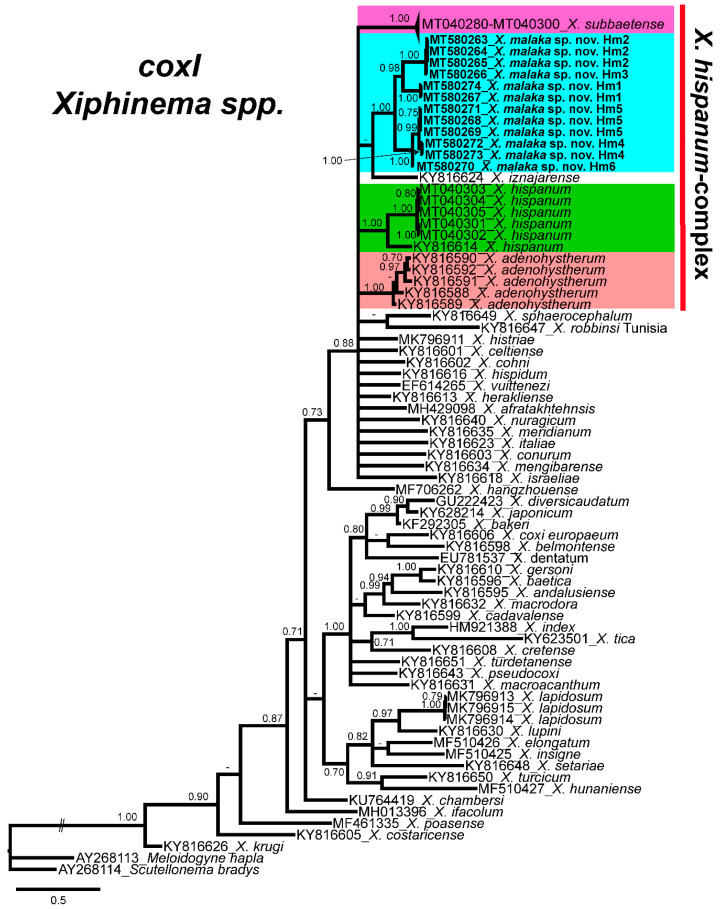
Phylogenetic relationships of *Xiphinema malaka* sp. nov. within the genus *Xiphinema*. Bayesian 50% majority-rule consensus trees as inferred from cytochrome c oxidase subunit I (*coxI*) mtDNA gene sequence alignments under the GTR + I + G model. Posterior probabilities more than 70% are given for appropriate clades. Newly obtained sequences in this study are in bold letters, and each colour is associated with each species of the complex.

Phylogenetic relationships among *Xiphinema* species inferred from analyses of *D2-D3* expansion domains of *28S* rRNA, *ITS1*, the partial *18S* rRNA and the partial *coxI* mtDNA gene sequences using BI are shown in Figure 3C, Figure 4, Figure 5 and Figure 6, respectively. The phylogenetic trees generated with the nuclear and mitochondrial markers included 136, 49, 65 and 95 sequences with 747, 1106, 1547 and 372 positions in length, respectively (Figure 3C, Figure 4, Figure 5 and Figure 6). The *D2-D3* tree of *Xiphinema* spp. showed a well-supported clade (PP = 1.00), including 10 species from morphospecies Groups 5 and 6, seven of them belonging to morphospecies Group 5 and three to Group 6, all of them reported from the Iberian peninsula, and included *X. malaka* sp. nov. (MT584052–MT584085). All other clades followed the same pattern as previous studies. *Xiphinema malaka* sp. nov. was phylogenetically related with *X. hispanum*, *X. celtiense* and *X. cohni* in a moderately supported clade (PP = 0.88), but clearly separate from all of them (Figure 3C).

Difficulties were experienced with alignment of the ITS1 sequences due to scarce similarity. Thus, only related sequences were used for phylogeny. The 50% majority rule consensus *ITS1* BI tree showed several clades low to moderately supported (Figure 4). *Xiphinema malaka* sp. nov. was phylogenetically related to *X. adenohystherum* and *X. iznajarense* in a moderately supported clade (PP = 0.92), but clearly separate from all of them (Figure 4).

The 50% majority rule consensus *18S* rRNA gene BI tree showed several major clades (Figure 5). Phylogenetic inferences based on *18S* suggest that *X. malaka* sp. nov. was related to other species of the *X. hispanum*-complex in a moderately supported clade (PP = 0.91), together with other species such as *X. barense*, *X. celtiense*, *X. cohni*, *X. gersoni*, *X. iznajarense*, *X. mengibarense*, and *X. sphaerocephalum* (Figure 5).

Finally, the phylogenetic relationships of *Xiphinema* species inferred from analysis of partial *coxI* gene sequences showed several clades that were not well defined (Figure 6). *Xiphinema malaka* sp. nov. (MT580263-MT580274) was phylogenetically related to *X. hispanum*-complex species in a low supported clade (PP = 0.65), but clearly separate from all of them (Figure 6).

### 2.5. Morphology and Morphometry of Xiphinema malaka *sp.* nov

**Figure 7:**
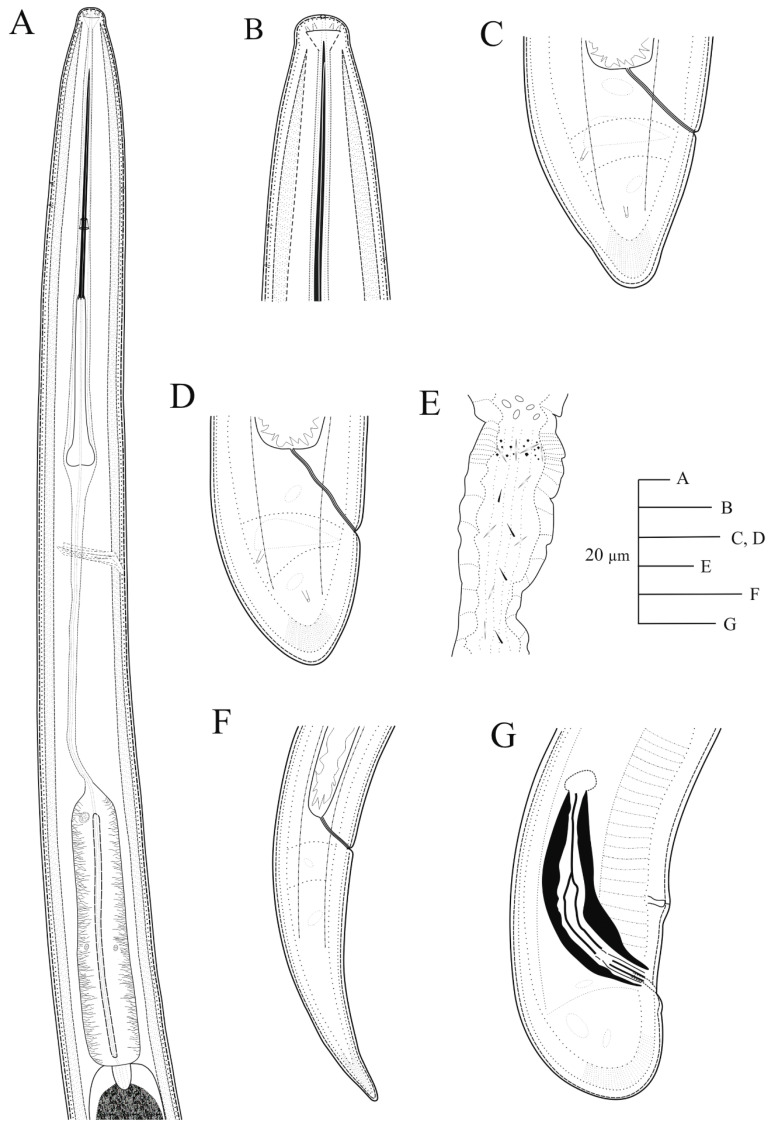
Line drawings of holotype for *Xiphinema malaka* sp. nov. (**A**), pharyngeal region; (**B**), detail of lip region; (**C**,**D)**, female tails; (**E**), detail of uterine pseudo Z-differentiation.; (**F**), tail of first-stage juvenile (J1); (**G**), male tail.

**Figure 8:**
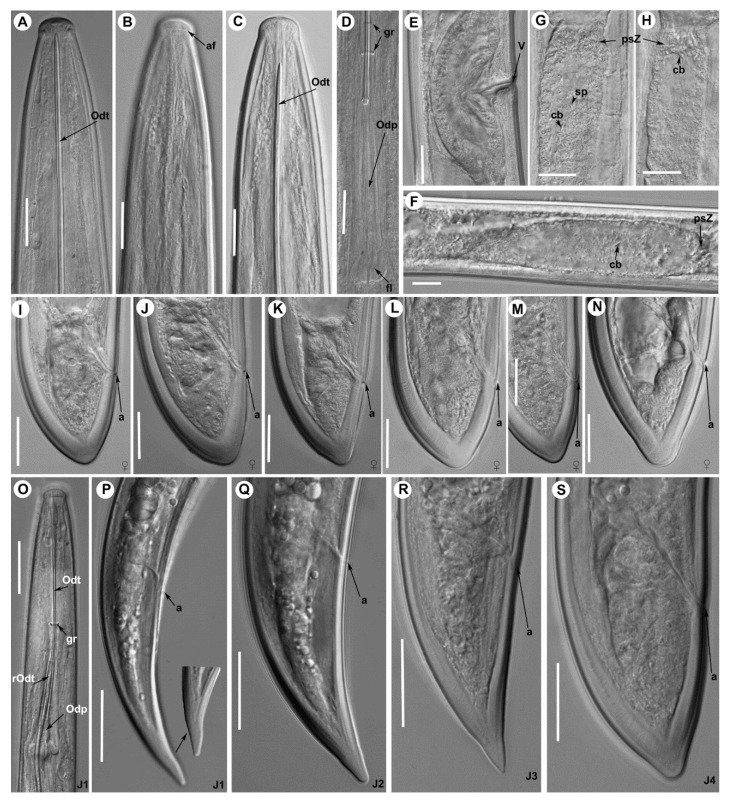
Light photomicrographs of *Xiphinema malaka* sp. nov. females holotype and paratypes: (**A**), anterior region holotype; (**B**,**C**) anterior regions paratypes; (**D**), detail of odontophore and guiding ring in holotype; (**E**), vulval region; (**F**–**H**), detail of female genital track showing Z-differentiation in holotype; (**I**), tail region of holotype; (**J**–**N**), tail region in paratypes; (**O**), detail of first-stage anterior region; (**P**–**S**), tail region of 1st, 2nd, 3rd and 4th stage juveniles. Abbreviations: a = anus; af = amphidial fovea; cb = crystalloid bodies; fl = odontophore flanges; gr = guiding ring; odp = odontophore; odt = odontostyle; psZ = pseudo-Z organ; rodt = replacement odontostyle; sp = spine; v = vulva. Scale bars: 20 μm.

**Figure 9:**
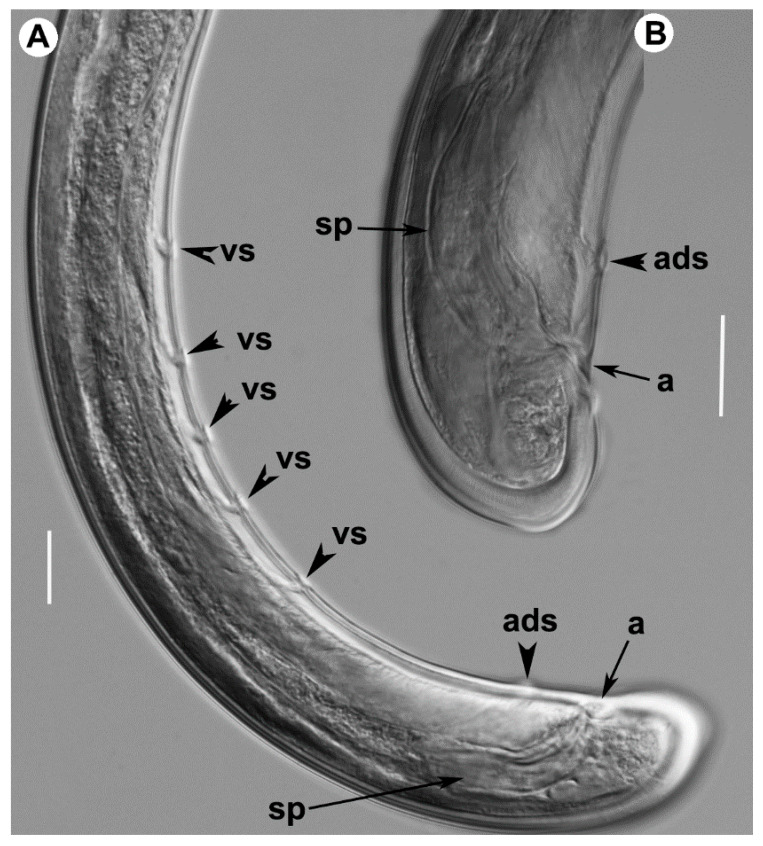
Light photomicrographs of *Xiphinema malaka* sp. nov. male: (**A**), posterior region; (**B**), detail of tail showing spicules. Abbreviations: a = anus; ads = adanal supplements; sp = spicules; vs = ventromedian supplements. Scale bars: 20 μm.

**Figure 10:**
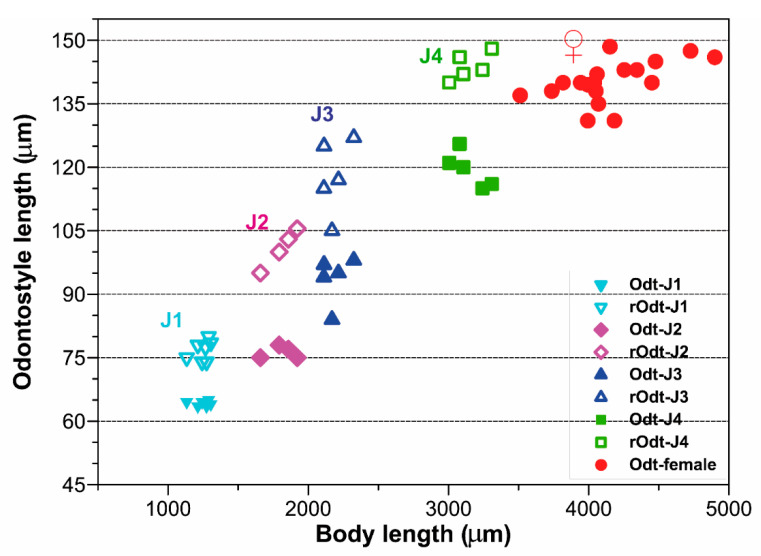
Relationship of body length to length of functional and replacement odontostyle (Ost and rOst, respectively) length in all developmental stages from first-stage juveniles (J1) to mature females of *Xiphinema malaka* sp. nov.

         ***Xiphinema malaka* sp. nov.**

http://zoobank.org/urn:lsid:zoobank.org:act: BDBF964D-71E8-4E4F-B61C-50C5A7C51083

(Figure 7, Figure 8, Figure 9 and Figure 10, Table 3 and Table 4)

#### 2.5.1. Material Examined

*Holotype.* Adult female was found in the rhizosphere of maritime pine (*Pinus pinaster* Aiton) at 1312 m a.s.l. from Canillas de Albaida, Málaga province, Spain (GPS: 36°52′21.81″ N; 3°55′41.00″ W) collected by A. Archidona-Yuste on 12 December 2019; mounted in pure glycerin and deposited in the nematode collection at Institute for Sustainable Agriculture (IAS) of Spanish National Research Council (CSIC), Córdoba, Spain (Slide number X-SA3-02).

*Paratypes.* Female and juvenile paratypes were collected from the same soil sample as the holotype (Table 3); mounted in pure glycerin and deposited in the Institute for Sustainable Agriculture (IAS) of the Spanish National Research Council (CSIC), Córdoba, Spain (Slide numbers X-SA3-03–X-SA3-08); one female at Istituto per la Protezione delle Piante (IPP) of Consiglio Nazionale delle Ricerche (C.N.R.), Sezione di Bari, Bari, Italy (X-SA3-011); one female at the USDA Nematode Collection (T-7474p).

*Additional material examined.* Additional nematode isolates were studied and characterized from the rhizosphere of maritime pine, black pine, cork oak and yellow broom at several localities at Málaga and Almería provinces (Table 4). Morphometric measurements were taken for 62 individuals, 40 females, one male and 21 juveniles from J1 to J4 from several localities in Málaga province, Table 3 and Table 4. Unfortunately, the scarce nematode isolate detected in the isolte of Tabernas (Almería) did not allow us to take measurements of adult females. 

*Type locality*. Canillas de Albaida, Málaga province, Spain (GPS: 36°52′21.81″ N; 3°55′41.00″ W); 1254 m above sea level (a.s.l.) collected by A. Archidona-Yuste on 12 December 2019. 

*Etymology.* The species epithet refers to the Phoenician word Malaka, the name of the province of Málaga where the species was found in several localities.

#### 2.5.2. Diagnosis. *Xiphinema malaka* sp. nov. 

Belongs to morphospecies Group 5 from the *Xiphinema* non*americanum*-group species [18]. It is characterized by a moderate long body (3.5–4.9 mm), assuming a J-shaped when heat-relaxed; lip region hemispherical, separate from the body contour by a depression, 14.0–15.0 μm wide; a relatively long odontostyle 131.0–148.5 μm; vulva located at 47.1–53.8% of body length; female reproductive system didelphic-amphidelphic having both branches about equally developed, pseudo Z-differentiation containing numerous small granular bodies, uterus tripartite with small crystalloid bodies and spines in low number and presence of prominent wrinkles in the uterine wall that may be confused with spiniform structures; female tail short convex-conoid on both sides, and bearing 3 caudal pores, ending in a rounded and broad terminus with a very small bulge at the end in some specimens; c’ ratio (0.9–1.0); male rare one individual out of 75 females. Four developmental juvenile stages were identified, the 1st-stage juvenile with tail elongate-conoid with characteristic subdigitate rounded terminus (c’ ratio 3.2–3.8). According to the polytomous key of Loof & Luc [18] and matrix codes sorted by Archidona-Yuste et al. [19], codes for the new species are (codes in parentheses are exceptions): A4-B23-C6-D6-E65-F4(5)-G3-H2-I3-J6-K2-L1. The DNA sequences of *D2-D3* expansion domains of *28S*, ITS1 rRNA, *18S* rRNA, and partial *coxI* were deposited in GenBank under the accession numbers MT584052-MT584085, MT584088-MT584099, MT584086-MT584087 and MT580263-MT580274, respectively.

#### 2.5.3. Description

*Female.* Body cylindrical, slightly tapering towards anterior end in a J-shape when heat relaxed. Cuticle with fine transverse striae visible in tail region, 3.2 ± 0.3 (3.0–3.5) μm thick at mid body but thicker just posterior to anus. Lateral chord 13.2 ± 2.5 (11.5–16.0) μm wide, occupying ca. 25% of corresponding body diam. Lip region hemispherical, slightly offset from body contour by a depression, 14.6 ± 0.4 (14.0–15.0) μm wide and 6.5 ± 0.6 (6.0–7.5) μm high. Odontostyle moderately long, 1.7–1.9 times longer than odontophore, the latter with well-developed flanges (13.0–15.5 μm wide). Guiding ring and guiding sheath variable in length depending on degree of protraction/retraction of stylet. Pharynx composed by a slender narrow flexible part 335–582 μm long, and a posterior muscular, cylindrical and expanded part with three nuclei. Terminal pharyngeal bulb variable in length, 112.0–149.0 μm long and 24.0–31.0 μm wide. Glandularium 110.0–129.0 μm long. Dorsal gland nucleus (DN) located at beginning of basal bulb (8.5–14.3%), ventrosublateral gland nuclei (SVN) situated *ca* halfway along bulb (52.3–67.9%) (position of gland nuclei calculated as described by Loof & Coomans [27]). Cardia conoid-rounded and variable in length, 12.0–15.0 μm long. Intestine simple, prerectum variable in size 471–516 μm long. Rectum 32.0–40.0 μm long ending in anus as a small rounded slit. Reproductive system didelphic-amphidelphic with two equally developed branches. Each branch composed of a short ovary 47–78 μm long, a reflexed oviduct 93–104 μm long with well-developed pars dilatata oviductus, a sphincter, a well-developed pars dilatata uteri, and a 254–286 μm long uterus with pseudo-Z differentiation containing numerous small granular bodies with small crystalloid bodies (6.0–12.5 μm long) and spines in low number, and presence of prominent wrinkles in the uterine wall that may be confused with spiniform structures (Figure 7 and Figure 8); a 35.5–47.0 μm long vagina perpendicular to body axis (having 28–32% corresponding body diam. ingrowth), ovejector well-developed 36.5–50.0 μm wide, pars distalis vaginae 16.8 ± 2.4 (13.0–19.5) μm long, and pars proximalis vaginae 24.3 ± 2.4 (20.5–27.5) μm long and 26.3 ± 1.0 (21.5–29.5) μm wide, and vulva as a transverse slit. Tail short, convex-conoid on both sides, and bearing three caudal pores, ending in a rounded and broad terminus, with a very small bulge at the end in some specimens (Figure 8).

*Male.* Extremely rare, only one male individual out of 75 female specimens was found in one sample near the type locality. Morphologically similar to female except for genital system and secondary sexual features. Male genital tract diorchic with testes containing multiple rows of different stages of spermatogonia. Tail short, convex-conoid with a broadly rounded terminus and thickened outer cuticular layer. Adanal supplements paired, preceded anteriorly by a row of five irregularly spaced ventromedians supplements. Spicules paired, dorylaimoid, moderately long and slightly curved ventrally, approximately 2.5 times longer than tail length; lateral guiding pieces more or less straight or with curved proximal end.

*Juveniles.* Four developmental juvenile stages were detected and distinguished by relative body length, odontostyle and replacement odontostyle length. The 1st-stage juveniles were characterized by the replacement odontostyle inserted into odontophore base (Figure 8). In all other stages, the replacement odontostyle was posterior to the flanges of odontophore in its resting position. The correlation between body length, replacement and functional odontostyle of the type population is given in Figure 10. Lip region in all juvenile stages looks similar to that in females. Other morphological characters similar to female, except for their size and immature sexual characteristics (developing genital primordium 16.0–87.0 μm long). The first-stage juvenile was characterized by a tail elongate-conoid with characteristic subdigitate rounded terminus (c’ ratio 3.2–3.8). Tail of other developmental stages becoming progressively shorter and wider after each moult (Figure 8).

#### 2.5.4. Remarks

According to the polytomous key by Loof & Luc [18] and matrix codes sorted by Archidona-Yuste et al. [6]: A (type of female genital apparatus), C (tail shape), D (c’ ratio), E (vulva position), F (body length), and G (total stylet length) (in this order of main features), *X. malaka* sp. nov. is closely related to *X. subbaetense* Cai, Archidona-Yuste, Cantalapiedra-Navarrete, Palomares Rius & Castillo [11], *X. hispanum* Lamberti, Castillo, Gomez-Barcina & Agostinelli [22], *X. adenohystherum* Lamberti, Castillo, Gomez-Barcina & Agostinelli [22], *X. cohni* Lamberti, Castillo, Gomez-Barcina & Agostinelli [22], and *X. sphaerocephalum* Lamberti, Castillo, Gomez-Barcina & Agostinelli [22].

*Xiphinema malaka* sp. nov. is morphometrically almost undistinguishable from *X. subbaetense* and *X. hispanum*, from the former can only be differentiated in females by a higher a ratio (65.6–99.8 vs. 49.0–70.0), a shorter odontophore (75.0–88.0 vs. 82.0–96.5 μm), narrower lip region (14.0–15.5 vs. 15.5–18.5 μm), higher c’ ratio in J1 (3.2–3.8 vs. 2.6–3.1, 2.7–3.1, respectively), and presence of male (very rare vs. absent) [11,20]. Morphologically can be differentiated from *X. subbaetense* and *X. hispanum* in pseudo-Z differentiation containing numerous small granular bodies vs. 4–5 granular bodies. It can be differentiated from *X. adenohystherum* by slightly shorter odontostyle (131.0–149.0 vs. 143.0–152.0 μm), longer tail (26.0–47.0 vs. 29.0–35.0 μm), and slightly higher a ratio (65.6–96.5 vs. 65.2–73.3). It can be differentiated from *X. sphaerocephalum* by its shorter odontostyle (131.0–149.0 vs. 143.5–168.0 μm), and shorter oral aperture-guiding ring distance (96.0–135.0 vs. 126.0–162.0 μm). Finally, *X. malaka* sp. nov. can be differentiated from *X. cohni* by its shorter odontostyle (131.0–149.0 vs. 149–174 μm), shorter oral aperture-guiding ring distance (96.0–135.0 vs. 137.0–161.0 μm), and higher c ratio (97.3–178.6 vs. 82.6–115.2). Nevertheless, it can be clearly separated by specific *28S* rRNA, ITS1 rRNA and *coxI* sequences.

## 3. Discussion

The primary objective of this study was to decipher the cryptic diversity of the *X. hispanum*-complex by applying an integrative taxonomical approaches on several new unidentified *Xiphinema* isolates from Málaga and Almería provinces (southern Spain), appearing morphologically and morphometrically indistinguishable from this species complex. Multivariate morphometric analyses proved to be useful tools for species delimitation within the genera *Longidorus* and *Xiphinema* [11,15,19,28]. These data support that *X. hispanum*-complex species comprise a model example of morphostatic speciation (genetic modifications not reflected in morphology and morphometry) [23,24], since independent approaches based on molecular analyses using ribosomal and mitochondrial sequences (haploweb and haplonet) revealed high levels of genetic diversity within these species complexes which clearly separated *X. malaka* sp. nov. from all other *X. hispanum*-complex species. These results, as well as those from previous studies, may suggest that *X. hispanum*-complex species comprises a *Xiphinema* endemic lineage, with members morphologically and morphometrically very similar, that have diversified in the Iberian peninsula, since no other records on these species have been reported outside this area [20,22,29].

Phylogenetic analyses based on three rDNA molecular markers (*D2–D3* expansion domains of *28S* rRNA gene, *ITS1* region and the partial *18S* rRNA) resulted in a general consensus of species phylogenetic positions for the majority of them, and were generally congruent with those given by previous phylogenetic analysis [6,11,19,30,31,32,33]. 

The results of this research support our hypothesis that biodiversity of Longidoridae in southern Spain is still not fully clarified and needs additional sampling efforts given the significant gaps in soil nematode biodiversity regarding the large number of undescribed species [34,35] and the hypothesis suggesting the Iberian Peninsula as a possible center of speciation for some groups of the family Longidoridae [6,15,36]. The recognition of this extraordinary cryptic diversity has a direct bearing on estimates of global nematode biodiversity and concepts of nematode biogeography. Regional endemicity in plant-parasitic nematodes has seldom been recognized and cosmopolitan distributions in nematodes, like other microscopic organisms, are reportedly common [37,38].

In summary, the present study confirmed the extraordinary cryptic diversity of *X. hispanum*-complex species in Andalusia and comprises a paradigmatic example of morphostatic speciation of dagger nematodes in southern Spain, which can be a potential explanation of the unusual high biodiversity within Longidoridae, considering Andalusia as a hot spot of biodiversity. However, additional similar intensive taxonomic studies are needed in other areas which can confirm this statement.

## 4. Materials and Methods

### 4.1. Nematode Isolates and Morphological Studies

No specific permits were required for the indicated fieldwork studies. The soil samples were obtained in public areas, forests and other natural areas and did not involve any endangered species or those protected in Spain, nor were the sites protected in any way.

A total of 62 individuals including 41 adults and 21 juvenile specimens from several localities in Málaga and Almería provinces (southern Spain) were used for morphological analyses (Table 1, Figure 1). Nematodes were surveyed during spring season in 2019 in natural ecosystems in Andalusia, southern Spain (Table 1). Soil samples were collected for nematode analysis with a shovel randomly selecting four to five cores at each point, and considering the upper 5–50 cm depth of soil that was close to the active plant root at each sampling spot. Nematodes were extracted from a 500-cm^3^ sub-sample of soil by centrifugal flotation [39] and a modification of Cobb’s decanting and sieving [40] methods. For morphometric studies, *Xiphinema* specimens were killed and fixed by a hot solution of 4% formalin + 1% glycerol, then processed in pure glycerin [41] as modified by De Grisse [42].

Specimens for light microscopy were killed by hot fixative using a solution of 4% formaldehyde +1% propionic acid and embedded in pure glycerine using Seinhorst’s [41] method. The morphometric study of each nematode isolate included morphology-based diagnostic features in *Xiphinema* (i.e., de Man body ratios), lip region width, amphid shape, oral aperture-guiding-ring, odontostyle and odontophore length and female tail shape [7]. For line drawings of the new species, light micrographs were imported to CorelDraw ver. X7 and redrawn. The light micrographs and measurements of each nematode isolate, including important diagnostic characteristics (i.e., de Man indices, body length, odontostyle length, lip region, tail shape, amphid shape and oral aperture-guiding ring; [7]) were performed using a Leica DM6 (Wetzlar, Germany) compound microscope with a Leica DFC7000 T digital camera. For the line drawings of the new species, CorelDraw software version X7 (Corel Corporation, London, UK) was used to redraw according to the selected light micrographs.

### 4.2. DNA Extraction, Polymerase Chain Reaction (PCR) and Sequencing

For molecular analyses, in order to ensure the selected nematodes for extracting DNA were from the same species, two live nematodes from each sample were temporary mounted in a drop of 1M NaCl containing glass beads (to avoid nematode crushing/damaging specimens) to ensure specimens conformed to the unidentified isolates of *Xiphinema*. Thus, 34 individuals collected from several sampling points in Andalusia were molecularly analyzed (Table 1). All necessary morphological and morphometric data, by taking pictures and measurements using the above camera-equipped microscope, were recorded. This was followed by DNA extraction from a single specimen and polymerase chain reaction (PCR) cycle conditions as previously described [6,15]. Several sets of primers were used for PCR. A partial region of the *28S* rRNA gene including the expansion domains D2 and D3 (*D2-D3*) was amplified by using the primers D2A (5′-ACAAGTACCGTGAGGGAAAGTTG-3′) and D3B (5′-TCGGAAGGAACCAGCTACTA-3′) [43]. The Internal Transcribed Spacer region 1 (*ITS1*) separating the *18S* rRNA gene from the 5.8S rRNA gene was amplified using forward primer *18S* (5′-TTGATTACGTCCCTGCCCTTT-3′) [44] and reverse primer rDNA1 5.8S (5′-ACGAGCCGAGTGATCCACCG-3′) [45]. A partial sequence of the *18S* rRNA gene (*18S*) was amplified as previously described [46] using primers 988F (5′-CTCAAAGATTAAGCCATGC-3′), 1912R (5′-TTTACGGTCAGAACTAGGG-3′), 1813F (5′-CTGCGTGAGAGGTGAAAT-3′), and 2426R (5′-GCTACCTTGTTACGACTTTT -3′. Finally, the portion of the cytochrome c oxidase subunit I gene (*coxI*) was amplified using the primers COIF (5′-GATTTTTTGGKCATCCWGARG-3′) and COIR (5′-CWACATAATAAGTATCATG-3′) [47]. The newly obtained sequences were deposited in the GenBank database under accession numbers indicated in Table 1 and on the phylogenetic trees.

PCR cycle conditions were one cycle of 94 °C for two min, followed by 35 cycles of 94 °C for 30 s, annealing temperature of 55 °C for 45 s, 72 °C for three min, and finally one cycle of 72 °C for 10 min. PCR products were purified after amplification using ExoSAP-IT (Affimetrix, USB products, High Wycombe, UK), quantified using a Nanodrop spectrophotometer (Nanodrop Technologies, Wilmington, DE, USA) and used for direct sequencing in both directions using the primers noted above. The resulting products were purified and run on a DNA multicapillary sequencer (Model 3130XL genetic analyser; Applied Biosystems, Foster City, CA, USA), using the BigDye Terminator Sequencing Kit v.3.1 (Applied Biosystems, Foster City, CA, USA), at the Stab Vida sequencing facilities (Caparica, Portugal). The newly obtained sequences were submitted to the GenBank database under accession numbers indicated in Table 1 and on the phylogenetic trees.

### 4.3. Species Delimitation via Multivariate Morphometric Analysis and Haplotype Networks Construction

The nine new *Xiphinema* isolates detected in this study were included in the *X. hispanum*-complex species group given the close relationships morphologically with *X. hispanum* as outlined above. An iterative analysis of morphometric and molecular data using two independent strategies of species delimitation was utilized to asses described and undescribed specimens and to determine species boundaries within this species complex.

Species delineation using morphometry was conducted with principal component analysis (PCA) in order to estimate the degree of association among species within the *X. hispanum*-complex [48]. PCA was based upon the following morphological characters: L (body length), the ratios a, c, c’, d, d’, V, odontostyle and odontophore length, lip region width and hyaline region length (Table 2) [6,7,13]. Prior to the statistical analysis, diagnostic characters were tested for collinearity [49]. We used the collinearity test based on the values of the variance inflation factor (VIF) method that iteratively excludes numeric covariates showing VIF values > 10 as suggested by Montgomery and Peck [50]. PCA was performed by a decomposition of the data matrix amongst isolates using the principal function implemented in the package psych [51]. Orthogonal varimax raw rotation was used to estimate the factor loadings. Only factors with sum of squares (SS) loadings > 1 were extracted. Finally, a minimum spanning tree (MST) based on the Euclidean distance was superimposed on the scatter plot of the *X. malaka* sp. nov.-specimens complex against the PCA axes. MST was performed using the ComputeMST function implemented in the package emstreeR [52]. All statistical analyses were performed using the R v. 3.5.1 freeware [53].

In order to detect distinct phylogenetic groups possibly representing separate species, haplotype networks (briefly, haplonet) were constructed to each of the two separate datasets, i.e., the *D2-D3* and *coxI*. Alignments were converted to the NEXUS format using DnaSP V.6 [54]; TCS networks [55] were applied in the program PopART V.1.7 [56]. Illustrations of networks were prepared using the program Adobe illustrator to add connecting curves between the haplotypes found co-occurring in heterozygous individuals [57].

### 4.4. Phylogenetic Analysis

Sequenced genetic markers in the present study (after discarding primer sequences and ambiguously aligned regions), and several *Xiphinema* spp. sequences obtained of GenBank, were used for phylogenetic reconstruction (Table 1). Outgroup taxa for each dataset were selected based on previous published studies [6,11,30,45,58]. Multiple sequence alignments of the newly obtained and published sequences were made using the FFT-NS-2 algorithm of MAFFT v. 7.450 [59]. Sequence alignments were visualized using BioEdit [60] and edited by Gblocks ver. 0.91b [61] in the Castresana Laboratory server (http://molevol.cmima.csic.es/castresana/Gblocks_server.html) using options for a less stringent selection (minimum number of sequences for a conserved or a flanking position: 50% of the number of sequences + 1; maximum number of contiguous no conserved positions: 8; minimum length of a block: 5; allowed gap positions: with half).

Phylogenetic analyses of the sequence data sets were based on Bayesian inference (BI) using MRBAYES 3.2.7a [62]. The best-fit model of DNA evolution was calculated with the Akaike information criterion (AIC) of JMODELTEST v. 2.1.7 [63]. The best-fit model, the base frequency, the proportion of invariable sites and the gamma distribution shape parameters and substitution rates in the AIC were then used in phylogenetic analyses. BI analyses were performed under a general time reversible, with a proportion of invariable sites and a rate of variation across sites (GTR + I + G) model for *D2-D3*, the partial *18S* rRNA, and the partial *coxI* gene, and under a transition model with a proportion of invariable sites and a rate of variation across sites (TIM2 +I + G). These BI analyses were run separately per dataset with four chains for 2 × 10^6^ generations. The Markov chains were sampled at intervals of 100 generations. Two runs were conducted for each analysis. After discarding burn-in samples of 30% and evaluating convergence, the remaining samples were retained for more in-depth analyses. The topologies were used to generate a 50% majority-rule consensus tree. Posterior probabilities (PP) were given on appropriate clades. Trees from all analyses were visualized using FigTree software version v.1.42 [64].

## Figures and Tables

**Table 1:** Isolates sampled for *Xiphinema malaka* sp. nov. from several localities of Málaga and Almería provinces (Southern Spain), and *Xiphinema adenohystherum* sequences used in this study.

Sample Code—Host-Plant Locality		*D2-D3* Haplotype	*coxI* Haplotype	*D2-D3*	*ITS1*	*18S*	*coxI*
***Xiphinema malaka*** **sp. nov.**							
**Maritime Pine (*Pinus pinaster* Aiton)**							
1. SA03-DF91	Canillas de Albaida (Málaga) ^a^	*D2-D3*-Hm3	*coxI*-Hm2	MT584052	MT584088	MT584086	MT580263
2. SA03-DF92	Canillas de Albaida (Málaga) ^a^	*D2-D3*-Hm10	*coxI*-Hm2	MT584053	-	-	MT580264
3. SA03-DF15	Canillas de Albaida (Málaga) ^a^	*D2-D3*-Hm10	*coxI*-Hm2	MT584054	-	-	MT580265
4. SA03-DF90	Canillas de Albaida (Málaga) ^a^	*D2-D3*-Hm10	-	MT584055	-	-	-
5. SA03-DF93	Canillas de Albaida (Málaga) ^a^	*D2-D3*-Hm10	-	MT584056	-	-	-
6. SA03-DF94	Canillas de Albaida (Málaga) ^a^	*D2-D3*-Hm10	-	MT584057	-	-	-
7. SA02-DF42	Canillas de Albaida (Málaga)	*D2-D3*-Hm12	-	MT584058	MT584089	-	
8. SA02-AU62	Canillas de Albaida (Málaga)	*D2-D3*-Hm12	-	MT584059	-	-	
9. SA05_DF16	Canillas de Albaida (Málaga)	*D2-D3*-Hm11	-	MT584060	MT584090	-	
10. SA05_DH96	Canillas de Albaida (Málaga)	*D2-D3*-Hm1	-	MT584061	-	-	-
11. SA05_DH97	Canillas de Albaida (Málaga)	*D2-D3*-Hm2	-	MT584062	-	-	-
12. SA05_DH98	Canillas de Albaida (Málaga)	*D2-D3*-Hm3	-	MT584063	-	-	-
13. SA05_DH99	Canillas de Albaida (Málaga)	*D2-D3*-Hm3	-	MT584064	-	-	-
14. SA06-DG12	Canillas de Albaida (Málaga)	*D2-D3*-Hm12	-	MT584065	MT584091	-	-
15. SA06-DG13	Canillas de Albaida (Málaga)	*D2-D3*-Hm5	-	MT584066	MT584092	-	-
16. SA06-DI01	Canillas de Albaida (Málaga)	*D2-D3*-Hm4	-	MT584067	-	-	-
17. SA06-DI02	Canillas de Albaida (Málaga)	*D2-D3*-Hm5	-	MT584068	-	-	-
18. SA06-DI03	Canillas de Albaida (Málaga)	*D2-D3*-Hm6	-	MT584069	-	-	-
19. SA06-DI04	Canillas de Albaida (Málaga)	*D2-D3*-Hm4	-	MT584070	-	-	-
20. SA07-AU46	Canillas de Albaida (Málaga)	*D2-D3*-Hm9	*coxI*-Hm3	MT584071	MT584093	MT584087	MT580266
21. SA07-AU47	Canillas de Albaida (Málaga)	*D2-D3*-Hm9	-	MT584072	MT584094	-	-
22. SA07-AU48	Canillas de Albaida (Málaga)	*D2-D3*-Hm9	-	MT584073	-	-	-
23. SA08-DF19	Canillas de Albaida (Málaga)	*D2-D3*-Hm2	*coxI*-Hm1	MT584074	MT584095	-	MT580267
**Black pine (*Pinus nigra*Arnold)**							-
24. SN01-DI10	Igualeja (Málaga)	*D2-D3*-Hm7	*coxI*-Hm5	MT584075	MT584096	-	MT580268
25. SN01-DE88	Igualeja (Málaga)	*D2-D3*-Hm3	*coxI*-Hm5	MT584076	-	-	MT580269
26. SN01-DF46	Igualeja (Málaga)	*D2-D3*-Hm3	*coxI*-Hm6	MT584077	-	-	MT580270
27. SN03-DE93	Igualeja (Málaga)	*D2-D3*-Hm7	*coxI*-Hm5	MT584078	MT584097	-	MT580271
**Cork oak (*Quercus suber* L.)**							
28. SN14-DF11	Monda (Málaga)	*D2-D3*-Hm8	-	MT584079	MT584098	-	-
29. SN14-DI12	Monda (Málaga)	*D2-D3*-Hm8	-	MT584080	-	-	-
30. SN14-DI13	Monda (Málaga)	*D2-D3*-Hm8	-	MT584081	-	-	-
31. SN14-DI14	Monda (Málaga)	*D2-D3*-Hm8	-	MT584082	-	-	-
32. SN14-DI15	Monda (Málaga)	*D2-D3*-Hm8	*coxI*-Hm4	MT584083	-	-	MT580272
33. SN14-DI16	Monda (Málaga)	*D2-D3*-Hm8	*coxI*-Hm4	MT584084	-	-	MT580273
**Yellow broom (*Cytisus scoparius* (L.) Link)**							
34. RAMB-AO44	Tabernas, Almería	*D2-D3*-Hm13	*coxI*-Hm1	MT584085	MT584099	-	MT580274
***Xiphinema adenohystherum***							
**Grapevine (*Vitis vinifera* L.)**							
406f	Villalba del Alcor, Huelva	-	-	-	MT584100	-	-
**Wild olive (*Olea europaea* L. subsp *europaea* var. *sylvestris*)**							
AR139	Aroche, Huelva	-	-	-	MT584101	-	-
**European holly (*Ilex aquifolium* L.)**							
Z137f	Arevalo de la Sierra, Soria	-	-	-	MT584102	-	-

^a^ Type locality (paratype specimens); (-) Not obtained or not performed.

**Table 2:** Eigenvectors and SS loadings of factors derived from nematode morphometric characters for *Xiphinema hispanum-*complex (*Xiphinema malaka* sp. nov., *Xiphinema adenohystherum*, *Xiphinema hispanum*, *Xiphinema subbaetense*).

	*Xiphinema hispanum*-Complex Principal Components ^a^		
Character ^b^	PC1	PC2	PC3
Body length (L)	−0.382	0.032	−0.003
a	−0.081	0.483	0.262
c’	0.009	0.137	0.774
d	−0.371	0.444	−0.100
d’	−0.318	−0.384	0.086
V	−0.170	−0.550	−0.046
Stylet	−0.523	−0.001	−0.015
Oa-gr	−0.440	0.198	−0.320
Hyaline region length	−0.334	−0.256	0.458
SS loadings	1.67	1.35	1.13
% of total variance	30.80	20.21	14.10
Cumulative % of total variance	30.80	51.01	65.1

^a^ Based on 41 female specimens of *Xiphinema malaka* sp. nov. from seven isolate samples, 25 female specimens of *Xiphinema subbaetense* from two isolate samples, eight female specimens of *Xiphinema adenohystherum* from a isolate sample, and 11 female specimens of *Xiphinema hispanum* from a isolate sample. Values of morphometric variables 1 to 3 (eigenvector > 0.458) are underlined. All isolates were molecularly identified and located at southern Spain. The c’ ratio was excluded by the multicollinearity test and then, it was not included in the multivariate analysis for the *Xiphinema hispanum*-complex; ^b^ Morphological and diagnostic characters according to Jairajpuri and Ahmad [7] with some inclusions. a  =  body length/maximum body width; c’ = tail length/body width at anus; d = anterior to guiding ring/body diam. at lip region; d’ = body diameter at guiding ring/body diameter at lip region; Oa-gr = Oral aperture-guiding ring distance; V = (distance from anterior end to vulva/body length) × 100.

**Table 3:** Morphometrics of paratypes for *Xiphinema malaka* sp. nov. from maritime pine (*Pinus pinaster* Aiton) at Canillas de Albaida (Málaga province) southern Spain ^a^.

		Paratypes				
Characters-Ratios ^b^	Holotype	Females	J1	J2	J3	J4
n	1	20	7	4	4	6
L (mm)	4.3	4.2 ± 0.36 (3.51–4.90)	1.25 ± 0.58 (1.13–1.31)	1.81 ± 0.11 (1.66–1.92)	2.19 ± 0.88 (2.11–2.33)	3.11 ± 0.16 (2.86–3.31)
a	73.6	74.2 ± 5.4 (65.6–82.2)	48.8 ± 2.1 (45.3–51.5)	43.2 ± 3.3 (40.8–48.1)	56.9 ± 2.0 (53.5–58.7)	64.8 ± 3.4 (62.1–70.0)
b	8.8	8.0 ± 0.7 (6.7–9.3)	5.1 ± 0.7 (4.4–6.1)	6.0 ± 0.8 (5.3–7.0)	5.9 ± 0.4 (5.5–6.3)	6.6 ± 0.5 (6.0–7.2)
c	117.4	110.6 ± 7.8 (97.3–126.3)	20.5 ± 1.1 (18.6–22.4)	28.5 ± 1.8 (26.8–31.0)	39.9 ± 1.6 (37.4–41.5)	67.4 ± 5.8 (60.2–75.4)
c’	0.9	0.9 ± 0.04 (0.9–1.0)	3.5 ± 0.2 (3.2–3.8)	2.4 ± 0.1 (2.3–2.6)	2.0 ± 0.1 (1.8–2.1)	1.3 ± 0.1 (1.2–1.4)
d	8.9	8.4 ± 0.7 (6.9–9.3)	5.4 ± 0.1 (5.3–5.6)	7.6 ± 0.1 (7.5–7.6)	7.3 ± 0.04 (7.3–7.4)	8.4 ± 0.5 (8.1–9.2)
d’	2.7	2.6 ± 0.1 (2.4–2.9)	2.0 ± 0.1 (1.9–2.1)	2.68 ± 0.02 (2.67–2.70)	2.4 ± 0.1 (2.3–2.5)	2.7 ± 0.1 (2.6–2.8)
V	50.1	50.2 ± 0.7 (49.2–51.5)	-	-	-	-
G1	13.7	13.6 ± 1.4 (11.1–15.8)	-	-	-	-
G2	12.5	11.6 ± 1.1 (9.9–13.5)	-	-	-	-
Odontostyle length	143.0	140.4 ± 4.7 (131.0–148.5)	64.1 ± 0.6 (63.5–65.0)	76.3 ± 1.5 (75.0–78.0)	93.6 ± 5.6 (84.0–98.0)	117.8 ± 4.0 (115.0–125.5)
Odontophore length	77.5	79.5 ± 1.9 (75.0–83.0)	40.6 ± 0.7 (40.0–42.0)	56.1 ± 1.5 (54.0–57.5)	59.9 ± 2.2 (57.5–63.0)	71.2 ± 2.0 (67.0–72.5)
Total stylet	220.5	219.9 ± 5.8 (206.0–229.0)	104.7 ± 1.1 (104.0–107.0)	135.8 ± 3.2 (132.5–140.0)	153.5 ± 5.9 (145.0–161.0)	189.0 ± 3.8 (187.0–197.0)
Replacement odontostyle	-	-	76.7 ± 2.4 (74.0–80.0)	100.9 ± 4.5 (95.0–105.5)	117.8 ± 8.8 (105.0–127.0)	143.9 ± 2.9 (140.0–148.0)
Lip region width	14.5	14.6 ± 0.4 (14.0–15.0)	9.4 ± 0.3 (9.0–9.5)	10.3 ± 0.4 (10.0–10.5)	11.1 ± 0.3 (11.0–11.5)	12.5 ± 0.4 (12.0–13.0)
Oral aperture-guiding ring	129.0	124.9 ± 8.8 (96.0–135.0)	51.6 ± 1.6 (50.0–54.0)	75.1 ± 3.6 (71.5–80.0)	81.80 ± 2.0 (80.0–84.0)	103.6 ± 4.5 (98.0–110.0)
Tail length	37.0	38.0 ± 2.3 (34.0–43.0)	60.8 ± 1.9 (57.5–64.0)	63.5 ± 1.7 (62.0–65.0)	54.8 ± 2.2 (53.0–58.0)	46.3 ± 2.6 (43.0–49.0)
J	12.0	11.9 ± 1.1 (10.0–13.5)	9.1 ± 0.6 (8.5–10.5)	13.8 ± 1.3 (12.5–15.0)	12.3 ± 0.3 (12.0–12.5)	10.5 ± 0.9 (9.5–11.5)

^a^ Measurements are in µm and in the form: mean ± standard deviation (range); ^b^ a = body length/maximum body width; b = body length/pharyngeal length; c = body length/tail length; c’ = tail length/body width at anus; d = anterior to guiding ring/body diam. at lip region; d’= body diam. at guiding ring/body diam. at lip region; V = (distance from anterior end to vulva/body length) × 100; J = hyaline tail region length; G1 = (anterior genital branch length/body length) × 100; G2 = (posterior genital branch length/body length) × 100.

**Table 4:** Morphometrics of *Xiphinema malaka* sp. nov. from several sampling points and localities at Málaga and Almería provinces, southern Spain^a^.

	Málaga Province								Almería Province
Host-plant Locality Sample Code	Maritime Pine (Canillas de Albaida) SA2	Maritime Pine (Canillas de Albaida) SA5	Maritime Pine (Canillas de Albaida) SA6		Maritime Pine (Canillas de Albaida) SA8	Black Pine (Iguaneja) SN1	Black Pine (Iguaneja) SN3	Cork oak (Monda) SN14	Yellow Broom Tabernas AO44
Characters-ratios ^b^	Females	Females	Females	Male	Female	Female	Female	Females	J4
n	1	4	9	1	1	1	1	2	1
L (mm)	4.6	4.5 ± 0.4 (4.0–5.0)	4.8 ± 0.2 (4.5–5.2)	4.8	4.3	4.3	4.2	(4.85, 4.92)	3.08
a	82.8	85.4 ± 11.0 (76.0–99.8)	87.2 ± 5.2 (79.6–96.5)	109.5	76.1	72.1	70.9	(82.0, 82.2)	70.0
b	9.9	9.0 ± 1.1 (8.0–10.0)	9.5 ± 0.7 (8.6–11.0)	9.2	9.0	12.2	8.1	(9.8, 10.3)	7.0
c	123.1	132.8 ± 15.2 (115.3–151.2)	164.7 ± 16.3 (133.2–178.6)	170.2	133.5	98.3	113.9	(103.2, 105.8)	65.5
c’	0.91	0.91 ± 0.03 (0.87–0.95)	0.78 ± 0.06 (0.72–0.89)	0.8	0.8	1.0	0.9	(1.0, 1.1)	1.3
d	8.5	8.2 ± 0.2 (8.0–8.4)	8.3 ± 0.3 (7.9–8.9)	7.6	7.6	8.1	9.4	(7.4, 7.9)	9.2
d’	2.6	2.48 ± 0.01 (2.47–2.50)	2.54 ± 0.13 (2.33–2.75)	2.3	2.5	2.6	2.8	(2.6, 2.7)	2.8
V or T	49.2	49.4 ± 0.6 (48.5–50.0)	49.8 ± 1.1 (47.8–51.5)	62.0	47.1	52.2	50.0	(51.8, 53.8)	-
G1	9.4	11.5 ± 0.8 (10.7–12.3)	12.1 ± 2.0 (9.9–13.6)	-	13.5	12.8	10.5	(9.2, 10.7)	-
G2	8.9	10.0 ± 0.7 (9.5–10.8)	11.4 ± 1.2 (10.1–12.3)	-	13.0	11.7	10.2	(8.1, 10.2)	-
Odontostyle length	135.5	137.0 ± 4.2 (132.0–141.0)	143.1 ± 2.2 (138.5–145.5)	144.5	131.0	134.5	136.0	(143.0, 149.0)	125.5
Odontophore length	79	80.9 ± 0.9 (80.0–82.0)	79.1 ± 2.7 (76.0–84.0)	78.0	83.0	80.0	80.0	(82.0, 88.0)	71.5
Total stylet	215	217.9 ± 4.9 (212.5–222.0)	222.2 ± 3.5 (215.5–227.5)	225.5	214.0	215.0	216.0	(231.0)	197.0
Replacement odontostyle	-	-	-	-	-	-	-	-	146.0
Lip region width	14.5	14.5 ± 0.4 (14.0–15.0)	14.7 ± 0.5 (14.0–15.5)	16.0	15.0	14.0	14.0	(15.0, 15.5)	14.0
Oral aperture-guiding ring	115	118.5 ± 2.1 (116.0–121.0)	122.5 ± 3.8 (119.0–129.0)	121.5	114.0	114.0	122.0	(115.0, 118.0)	110.0
Tail length	37	33.6 ± 1.1 (32.5–35.0)	29.2 ± 2.9 (26.0–34.5)	28.0	32.5	44.0	37.0	(46.5, 47.0)	47.0
J	12.0	10.3 ± 0.4 (10.0–10.5)	10.5 ± 2.0 (8.0–13.0)	8.0	8.5	12.0	11.0	(13.0)	12.0
Spicules	-	-	-	64.0	-	-	-	-	-
Lateral guiding pieces	-	-	-	26.5	-	-	-	-	-

^a^ Measurements are in µm and in the form: mean ± standard deviation (range); ^b^ a = body length/maximum body width; b = body length/pharyngeal length; c = body length/tail length; c’ = tail length/body width at anus; d = anterior to guiding ring/body diam. at lip region; d’= body diam. at guiding ring/body diam. at lip region; V = (distance from anterior end to vulva/body length) × 100; J = hyaline tail region length; G1 = (anterior genital branch length/body length) × 100; G2 = (posterior genital branch length/body length) × 100.
